# The timid invasion: behavioural adjustments and range expansion in a non-native rodent

**DOI:** 10.1098/rspb.2023.0823

**Published:** 2023-07-26

**Authors:** Jana A. Eccard, Valeria Mazza, Celia Holland, Peter Stuart

**Affiliations:** ^1^ Animal Ecology, Institute for Biochemistry and Biology, University of Potsdam, Potsdam, Germany; ^2^ Department of Zoology, School of Natural Sciences, Trinity College Dublin, College Green, Dublin 2, Ireland; ^3^ Department of Biological and Pharmaceutical Sciences, Munster Technological University, Clash, Tralee, Ireland

**Keywords:** animal personality, spatial sorting, biological invasions, *Myodes glareolus*, risk-taking, small mammals

## Abstract

Animal behaviour can moderate biological invasion processes, and the native fauna's ability to adapt. The importance and nature of behavioural traits favouring colonization success remain debated. We investigated behavioural responses associated with risk-taking and exploration, both in non-native bank voles (*Myodes glareolus*, *N* = 225) accidentally introduced to Ireland a century ago, and in native wood mice (*Apodemus sylvaticus*, *N* = 189), that decline in numbers with vole expansion. We repeatedly sampled behavioural responses in three colonization zones: established bank vole populations for greater than 80 years (2 sites), expansion edge vole populations present for 1–4 years (4) and pre-arrival (2). All zones were occupied by wood mice. Individuals of both species varied consistently in risk-taking and exploration. Mice had not adjusted their behaviour to the presence of non-native voles, as it did not differ between the zones. Male voles at the expansion edge were initially more risk-averse but habituated faster to repeated testing, compared to voles in the established population. Results thus indicate spatial sorting for risk-taking propensity along the expansion edge in the dispersing sex. In non-native prey species the ability to develop risk-averse phenotypes may thus represent a fundamental component for range expansions.

## 1. Introduction

Behaviour enables animals to cope with novel environments and challenges [1]. Behaviour allows, for example, adjustments to habitats in which a species had not evolved [2,3]. Recent growth in the study of inter-individual variation in behaviour [4–6], however, revealed that behaviour is not entirely flexible [5], and that individuals differ consistently in behavioural traits within a population [7]. Further, this consistent variation has fitness consequences [8,9]. For the success of biological invasions of animals, two contrasting concepts have been suggested: behavioural invasion syndromes and behavioural flexibility [10], i.e. the ability to respond quickly to altered environmental conditions. Behavioural syndromes are sets of consistent inter-individual differences in behaviour that occur together [5]. Traits increasing colonization success may include high dispersal tendency, high foraging efficiency, and risk-taking in new environments (for review, see [11]).

Colonization of animals to previously unoccupied areas can be distinguished into two separate spatiotemporal processes: expansion and settlement, during which selective pressures on traits related to dispersal, e.g. morphological trait distribution within the population, can shift [12,13]. This process can lead to spatial sorting of populations being adapted to dispersal at the edge, and adapted to high intraspecific competition in established populations [14]. While this was shown for morphological traits related to movement [15], the evidence for spatial sorting in behavioural traits remains equivocal, both for existence of behavioural sorting itself [16,17] as well as for the behavioural types favourable in different spatial zones of a non-native expansion [16,18,19].

**Figure 1.**
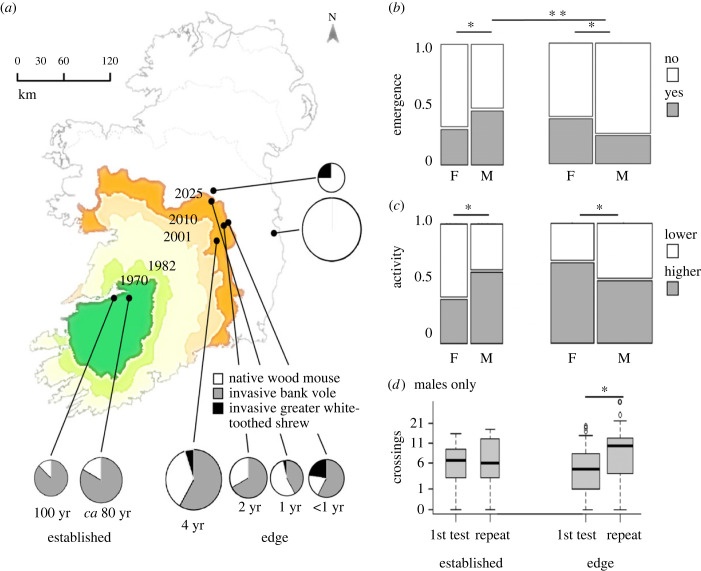
(*a*) Colonization history of the bank vole population introduced a century ago to Ireland (dark green area). Map lines delineate boundaries of modelled range expansion according to White *et al*. [22]. Rodent populations were sampled at 8 sites (black dots); size of corresponding pie chart indicates population density during the study, ranging from 9 (smallest) to 53 (largest) small rodents per 100 trap nights. Shading of pie chart depicts local species composition at capture. (*b–d*) Boldness and exploration of non-native bank voles in established (near source location of introduction) and edge zone of the colonization. (*b*) Proportion of emerging animals within 5 min in a dark–light test; (*c*) exploration of an open field arena indicated by activity type (bimodal distribution converted into lower activity/higher activity with cut-off at 50%); and (*d*) exploration of an open field arena indicated by crossing into its centre. Shown are interactive effects of colonization zone × sex of the tested animal (*a,b*) and habituation of males to the test situation, i.e. first versus repeated testing (*c*) (electronic supplementary material, tables S2–S4). Widths of bars and boxes indicate relative sample size.

To understand how colonization occurs, and which candidate traits may favour expansion in a non-native environment, we need to monitor an expansion in real time. An ongoing rodent colonization of Ireland by a continental vole species [20], that started a century ago and is currently covering more than half of Ireland, offers a unique opportunity to study colonization processes in an ecologically homogeneous area with a single, poorer, competitor (the wood mouse, *Apodemus sylvaticus*), and a genetically homogeneous invader population from a single origin. The bank vole (*Myodes glareolus*) is believed to have been introduced to Ireland at Foynes port, in the west of Ireland, from Germany during the construction of a hydroelectric dam in 1919/1920 [21]. After an initial lag period during establishment the rate of spread has been approximately 2.5 km yr^−1^, with no eradication programme applied [22]. Therefore, the colonization gradient can be divided into different zones: established populations being the area around Foynes where voles were introduced in the 1920s, the expansion edge being areas furthest away from the introduction site where voles are now found but were found to be unoccupied in earlier recent studies, and pre-arrival zone were voles have not arrived yet. We investigated behavioural traits of both the non-native bank vole and the single native small rodent woodland species the wood mouse at 8 site replicates (figure 1*a*) with comparable woodland vegetation in the different colonization zones, testing 414 rodents in 533 behavioural tests. At the established zone (2 sites) voles have been present for 80–100 years [20] and coexist with relatively low numbers of mice; at the edge (4 sites) mice and voles coexist both in relatively even numbers; and at the pre-arrival zone (2 sites) mice were found in relatively high numbers (figure 1*a*, and electronic supplementary material, table S1) but no voles.

We focused on two aspects of behaviour which are likely to influence individuals' success during the various stages of colonization (*sensu* [23]), i.e. the amount of exploration in an unfamiliar environment and the propensity to take risks, or boldness [24]. Bolder individuals are expected to reap greater rewards in terms of mates and/or resources, and thus to have better chances to navigate novel or potentially hostile environments (e.g. [2,23]). Inter-individual variation in exploration predicts dispersal tendency and space use in several species (e.g. [24,25]), and is also suggested to facilitate range expansion in non-native habitats (e.g. [26]). We hypothesized that voles at the edge of the expansion would differ in the mean expression of personality traits related to risk-taking and exploration, as well as in the level of flexibility of those traits compared to individuals of established populations. We predicted that (a) voles at the expanding edge would behaviourally differ from voles in established populations. They could take either more risks (e.g. [27]) or fewer risks while continuously facing new challenges reflecting a reactive coping style with shy and ‘slow–thorough’ explorers [28–30]. We further predicted that (b) voles at the expansion edge would habituate faster to the novel test situation compared to those from established zones, as they are generally more likely to experience unpredictable conditions and unsuitable habitats. Rodents in urban areas, for example, showed more bold and more flexible behaviour in response to these challenges [3,31]. Further, knowing that in many species males and females differ in space use and dispersal tendency [32–34] we assumed that the sexes may be exposed to differential selection pressures during the process of population expansion. With space use being closely related to behavioural traits [9,25], selection on space use and dispersal traits may produce spatial sorting patterns in personality traits differing among males and females. We therefore (c) investigated interactions of sex and expansion zone on the behaviour of the phenotyped individuals. Since biological invasions have the deepest impact on native fauna/biodiversity, we additionally investigated whether the native wood mice would show a behavioural adjustment in response to the presence of bank voles. We investigated the behaviour of mice at sites with established bank vole populations, bank vole edge populations and additionally at pre-arrival sites, which were still vole-free. Although considered native (i.e. present in Ireland for more than 500 years) wood mice were introduced to Ireland probably a millennium ago [35], allowing competitive release from bank voles and other forest rodents they co-evolved with in Continental Europe. The addition of an introduced, non-native competitor can alter personality traits in the native fauna [36], shifting niches to allow coexistence [37] with a novel competitor. Meanwhile, since wood mice numbers in Ireland decline with persistence of voles [38,39], we expected that there are no behavioural responses of mice mitigating effects of competition with voles, and thus mice behaviour should not differ among colonization zones.

## 2. Methods

### (a) Study locations

Rodent populations were studied in summer 2019 when the bank voles' expansion edge had migrated 200–250 km east of the established population, at 2 sites near the introduction location, 4 sites near the distribution edge, and 2 pre-arrival sites (figure 1*a*, table 1 and electronic supplementary material, table S1). Sampling locations in forest fragments were selected based on Stuart *et al*. [39]. Forest in Ireland covers only 11% of the country [40] but forest fragments in Ireland are probably well connected for forest small mammals through hedges, stonewalls and vegetation along ditches. Sites were characterized by broad-leaved trees, with leaf litter and ivy as ground cover (for vegetation information, see electronic supplementary material, table S1).

**Table 1.** Information on study sites in Ireland, relative to the population expansion of the non-native bank vole (expansion zones, site description, and rodent population). Rodent density is indexed by individuals per 100 trap nights (excluding recaptures of marked animals). Predator observations combine field observation, trap captures, and camera trap pictures (4 cameras set for two nights at all sites; plus 20 spy box cams set at ground level for 3 nights at selected sites). Tests include behavioural tests (emergence test and open field test) on first and repeated captures.

zone (site)	vole presence (years)	distance from vole introduction (km)	size of forest fragment (ha)	main tree species	mammalian predator(s) observed	study days (visits)	trapping grids	trap nights	rodents/100 trap nights	voles (%)	tests
establ (Foy)	approximately 100	5	6.9	ash, beech	rats, dogs, mustelid	6 (2)	2	288	19	87	86
establ (CC)	approximately 80	12	10.9	beech	no observ.	4 (2)	2	192	37	83	58
edge (MA)	4	146	94.5	ash	mustelid, dog	5 (2)	2	182	50	62	116
edge (KT)	2	156	11.6	ash	rats, cats, mustelid	6 (2)	1	288	24	67	86
edge (LD)	1	157	105.8	mixed deciduous	no observ.	6 (2)	2	288	17	44	89
edge (LM)	<1	165	2.9	oak/beech/willow	cats	2 (1)	3	124	16	75	19
pre (MF)	absent	160	2.5	beech	rats	3 (2)	2	186	6	0	12
pre (KS)	absent	205	62.1	ash	rats, dogs, badger	3 (2)	2	144	42	0	67

### (b) Trapping procedures

Rodents were trapped in single-capture Longworth traps (Penlon Ltd, Oxford, UK) which were pre-baited with seeds from commercial bird feed for 2 nights prior to trapping. At each site, 24 traps were set in lines with 10 m spacing between them. Traps were baited and set overnight and checked and deactivated in the morning. Animals were kept inside their traps in the shade until testing, with replenished food and pieces of carrots and cucumber as water sources. Trapping was not conducted during rainy days. Sites were visited for two trapping sessions within 4 days and re-visited after 2–4 weeks to allow repeated testing on recaptured individuals. A general problem of capture methods that require active participation of individuals to enter the trap may be a bias towards individuals bold enough to enter the trap on the first night. However, trappability was found to be not repeatable and not connected to personality in several species of small mammals [41]. We are confident that pre-baiting and capturing over several subsequent days minimized potential bias concerning trap avoidance. Further, finding behavioural differences in the trappable part of the population among zones would be a conservative estimate of differences among entire populations.

### (c) Recapture rates and population structure

We captured non-native bank voles, native wood mice, and in small numbers new non-native species [38], the greater white toothed shrew (*Crocidura russula*) on four of the edge and pre-arrival sites. Proportions of captured individuals by species are shown for each site in figure 1*a*. As in Stuart *et al*. [39], the proportion of voles relative to mice was higher in the established zone than at the expansion edge (figure 1*a*). Across all sites, we captured and tested 196 individual bank voles, of which 67% were adults and 54% of adults were male. Recapture rate of voles was 35% of animals within initial capture series of 2–4 days, and 27% after 2–4 weeks. Behaviour of all recaptured animals was tested. We captured 237 wood mice across sites; however, a third of the agile mice escaped unhandled and unmarked during or after the test procedure. 80% of identified mice were adult and 58% of adults were male. 32% of animals were identified recaptures during the first and 42% during the second visit. Rodent captures (mice plus voles, corrected for trap nights) and demography of populations (sex, age, body weight, probability of recapture (as surrogate of population turnover rate)) of each species did not vary among zones of the colonization by voles (mixed effect models based on individual captures, including the individual ID and the research visit ID; electronic supplementary material, table S3). With the focus of the present study on behaviour, we report the population background of the sampled animals here as part of the methods.

### (d) Assessing risk-taking and exploration

For behavioural testing, we adopted two standardized behavioural tests that are commonly used in animal personality studies of small mammals to assess boldness and spatial exploration in a novel environment. The set-up combined the dark–light test [42] and the open-field test [43], and was adjusted to be executed under field conditions without prior handling of animals (e.g. [3,25]). Test arenas were always set in shady locations or under canvas roofs to avoid direct sunlight, shade patterns and open sky above the arena. At the end of each test, the arenas were cleaned with ethanol 70%. All tests were conducted between 10.00 and 18.00, under natural light conditions.

#### (i) Dark–light test

Here, we define risk-taking as the behaviour expressed in a potentially dangerous situation, and boldness as consistent individual differences in risk-taking behaviour (e.g. [6]). We assessed boldness by measuring individuals' willingness to emerge from a dark covered shelter into an open, illuminated, and empty arena, which is perceived as dangerous for a rodent species with a plethora of predators (e.g. [44,45]). An opaque plastic tunnel (15 cm inner diameter, 30 cm long) was connected to an opening in the circular arena at ground level, blocked by a wooden door. Shortly before the tests, the animal was carefully transferred from the trap into a shuttle tube (15 cm outer diameter, 30 cm long, open at one side and blocked by a cylindrical sponge of the same diameter on the other) avoiding touching or shooing. The shuttle tube with the animal inside was then inserted with its full length into the plastic tunnel. After 1 min of acclimatization to the dark tunnel, the door leading into the arena was opened and we measured the rodent's latency to emerge (emergence) from the dark tunnel into the arena with its full body (without the tail). If animals did not emerge from the tunnel within 5 min (66% of tests in voles and 24% in mice), we slowly reduced the space in the tube by pushing in the soft, cylindrical sponge in order to gently displace the animal without touching it. To reduce potential observer effects, we carried out observations from behind the emergence tube, so animals could not spot the observer before or while emerging.

#### (ii) Open field test

Our operational definition for exploration was the consistent individual differences in exploratory behaviour in the context of space and in connection to movement (e.g. [26,38,39]. We quantified exploration by measuring the proportion of time each individual spent moving in the novel space of an experimental arena (activity), and also how often they crossed into its central part (crossings). We used round, plastic, foldable arenas (1.20 m diameter, 40 cm high) as portable test set-ups [46]. Round arenas prevent animals retreating to corners. The central part of the arena (90 cm diameter) was more exposed (open field) and could be perceived as potentially dangerous by the animal, compared to the area (15 cm wide) nearby the wall (e.g. [44,45]). We quantified exploration activity by measuring the proportion of time each individual spent moving in the novel space of an experimental arena, and also how often they crossed into its central part, which should be perceived as being even more exposed and potentially dangerous. Arenas were covered with mesh to prevent wood mice from jumping out, which also gave a visual barrier to reduce potential observer effects. Further, we sat very still and avoided bending over the arena, or staring at the animal, while recording the behaviour on paper.

The open field test started as soon as the animals entered the arena from the dark tunnel of the dark–light test, with the full body (without the tail). The door leading to the dark tube was closed quietly by the observer, or, if the animal was pushed out, the tube remained blocked by the sponge. For 5 min after emergence, we counted the number of 10 s intervals spent moving in the arena (activity), and the number of crossings of the line dividing the wall area and the central part of the arena with the whole body (crossings). After testing, animals were removed from the arena by offering a hiding place, either by pulling back the sponge in the tube or by placing their familiar trap in front of them.

After the behavioural tests, animals were sexed and weighed, marked by an individual fur cut if not already marked, and photographed for later identification.

### (e) Statistical analyses

#### (i) Assessing the repeatability of inter-individual variation in behaviour

As a precondition to our comparisons of individual behaviour among colonization zones, we first challenged the assumption that behavioural traits are consistent within and among individuals and are thus a useful representation of an animal's behavioural type or personality [6]. Repeatability (R) is a population-specific metric used to quantify among-individual phenotypic differences across time or contexts relative to the variation in the population. We calculated repeatability with the R package rptR [47], using individual ID as a random factor in generalized linear mixed models ((G)LMMs) adjusting for repeated testing (first versus repeat) [35]. We analysed 193/120 first/repeated tests for voles and 91/56 for mice. Seventy-two behavioural tests for mice were removed from the repeatability analysis due to the agility of mice: after a single animal had escaped unmarked at a particular site, it was no longer possible to identify first captures with certainty.

#### (ii) Modelling individual behaviour in different zones of the colonization

We investigated inter-individual differences in boldness and exploration between rodents at the expansion edge and at the established zone using GLMMs (with R package lme4). We added zone of the colonization (2 zones for bank voles, 3 zones for mice (figure 1*a*)), sex (male versus female), and test repeat (first test versus repeated tests to account for habituation to testing conditions) as fixed effects to model behavioural variables. We used a combination of siteID and visitID (23 combinations) as a random effect, to account for (a) temporal variation (daytime, weather, season, involved team members) and spatial (sites) variation in the data and (b) dependence of tested individuals from the same place and time. We used animal ID as a second random factor to control for repeated testing of the same individuals (a third of the tests were repeats). Initial models included two-way interactions of the main effects. Interactions were removed if not significant. We report log-likelihood tests (Wald's chi^2^) in the text and show effect sizes in tables and provide R2 to indicate variation explained without (marginal) and with (conditional) random factors [48].

We used all available mouse data (including animals that escaped before ageing, sexing and individual identification) for a model comparing between zones. We conducted additional analysis including zone and individual information (sex, repeated testing) with a reduced dataset on identified mice only (see electronic supplementary material, table S4, for sample sizes for different variables). Since juvenile mice did not move in the open-field test, we stopped testing them and did not analyse the behavioural data initially collected on juveniles.

In the vole dataset, variables were zero inflated (29% of voles never crossed into the centre of the arena) or bimodal (voles left the dark space either immediately or not at all during the 5 min; activity in the open field had peaks at less than 10% or greater than 90% activity) and were therefore analysed as binomials. Within the subset of trials where animals had actually crossed into the centre of the arena, we also analysed the number of crossings.

## 3. Results

We quantified risk taking propensity and exploration of 225 voles and 189 adult mice in 533 behavioural tests, using personality tests established for behavioural phenotyping of small rodents under field conditions [25]. Not all data could be used in all analyses since some animals managed to escape before marking or sexing (electronic supplementary material, data S1).

We found that for both rodent species the recorded behaviour varied consistently among individuals, in relation to the variance measured among conspecifics (see repeatability scores for emergence, crossings and activity in electronic supplementary material, table S2), allowing us to investigate adaptations at the phenotypic, individual level. Boldness, i.e. the emergence in the dark–light test, and one exploration measure, i.e. the number of crossings into the central arena in the open field test, were repeatable (in tendency for emergence in mice), while activity was not (electronic supplementary material, table S2).

Colonization zone explained the behavioural responses of bank voles in an interaction with sex of the tested animal (table 2 and *post* *hoc* tests, table 3). At the expanding edge of the colonization, males were less likely to emerge from the dark into the open test arena (24% emerged), compared to males at the established zone of the population (49%), and compared to the females at the edge (38%), while in the established populations, male emergence was similar to females (29%; GLMM, interaction zone × sex log-likelihood test: *χ* = 7.2, *p* = 0.008, *post hoc* tests, table 3, figure 1*b*). Bank vole males at the expansion edge were less explorative than females, while males’ exploration was higher than females in established populations (interaction zone × sex, *χ* = 8.7, *p* = 0.003, figure 1*c*). In established populations, sex differences were expressed in the opposite direction: a larger proportion of males were bolder and more explorative (*post hoc*, *z* > 2.0, *p* = 0.044), while at the expansion edge, males were more risk-averse and less explorative (*z* < −2.2, *p* < 0.029) compared to the females in the respective zone (interaction of zone × sex: emergence: *χ*^2^ = 7.2, *p* = 0.007; activity *χ*^2^ = 8.7, *p* = 0.003; number of crossings into the centre of the arena: *χ* = 11.4, *p* < 0.001; figure 1, table 3).

**Table 2.** Bank voles: full models of behavioural variables emergence, crossings and activity compared between two main colonization zones (established and edge), sexes (M and F), and habituation to the test (recapture yes/no); analysed with a GLMM (package lme4). Brackets indicate sample sizes (no. of tests/random effect individuals/random effect test occasions). Given are effect sizes (estimate) and standard error (s.e.) and sample sizes; *R*_marg_ and *R*_cond_ indicate variation explained without (marginal) and with (conditional) random effects. Significant effects are marked in bold font.

variable	factors	estimate	s.e.	d.f.	*z*/*t*	*p*	*R*_marg_	*R*_cond_
emergence (binomial)	intercept	−1.54	0.53		−2.92	0.003	0.17	0.59
	colonization zone (edge)	0.61	0.59		1.04	0.298		
	**sex (M)**	**1.37**	**0.58**		**2.35**	**0.019**		
	recapture (yes)	0.66	0.35		1.88	0.06		
(291/202/26)	**colonization zone (edge) × sex (M)**	**−2.33**	**0.77**		**−3.04**	**0.002**		
crossings (Poisson)	intercept	1.53	0.23		7.15	< 0.001	0.02	0.33
	colonization zone (edge)	0.3	2423		1.25	0.221		
	recapture (yes)	−0.19	0.16		−1.21	0.226		
	**sex (M)**	**0.59**	**0.18**		**3.25**	**0.001**		
	**colonization zone (edge) × sex (M)**	**−0.79**	**0.23**		**−3.28**	**< 0.001**		
(203/150/24)	**recapture (yes) × sex (M)**	**0.45**	**0.19**		**2.38**	**0.017**		
activity (binomial)	intercept	−0.6	0.44		−1.33	0.18	0.31	0.53
	**colonization zone (edge)**	**1.29**	**0.53**		**2.43**	**0.015**		
	**sex (M)**	**0.91**	**0.43**		**2.11**	**0.035**		
	recapture (yes)	0.48	0.28		1.74	0.082		
(290/199/26)	**colonization zone (edge) × sex (M)**	**−1.64**	**0.55**		**−2.99**	**0.003**

**Table 3.** *Post hoc* tests for bank vole behaviour. Disentangling interactions from table 2 by calculating simple effects within factor levels of the respective other factor; using GLMM as in table 2. Brackets indicate sample sizes nested within random effects (no. of tests/no. of individuals/no. of test occasions). Given are the standard error (s.e.). Marginal and conditional *R*^2^ values were similar to given for full models (table 2). Interactions were removed if not significant in any of the *post* *hoc* analyses. Significant effects are marked in bold font.

data subset	factors	estimate	s.e.	*z*/*t*	*p*
**emergence (binomial)**					
**males**	intercept	−0.3	0.43	−0.71	0.478
(162/103/21)	colonization zone (edge)	**−1**.**56**	**0**.**61**	**−2**.**58**	**0**.**010**
	recapture (yes)	0.7	0.45	1.56	0.118
**females**	intercept	−1.44	0.67	−2.14	0.032
(129/99/21)	colonization zone (edge)	0.41	0.61	0.67	0.501
	recapture (yes)	0.69	0.6	1.14	0.253
**edge**	intercept	−0.96	0.39	−2.44	0.015
**(179/125/18)**	sex (M)	**−1**.**01**	**0**.**46**	**−2**.**21**	**0**.**027**
	recapture (yes)	**0**.**87**	**0**.**43**	**2**.**02**	**0**.**044**
**established**	intercept	−1.84	0.81	−2.29	0.022
(112/79/15)	sex (M)	**1**.**78**	**0**.**7**	**2**.**55**	**0**.**011**
	recapture (yes)	0.24	0.54	0.44	0.658
**crossings (Poisson)**					
**males**	intercept	1.45	0.24	5.96	<0.001
(109/75/20)	colonization zone (edge)	−0.04	0.3	−0.14	0.891
	recapture (yes)	0.24	0.22	1.06	0.289
	colonization zone (edge) × recapture (yes)	0.31	0.27	1.13	0.257
**females**	intercept	1.5	0.2	7.47	<0.001
(80/66/20)	colonization zone (edge)	0.29	0.23	1.3	0.193
	recapture (yes)	−0.08	0.27	−0.28	0.78
	colonization zone (edge) × recapture (yes)	0.18	0.3	0.59	0.557
**edge**	intercept	1.82	0.14	12.84	<0.001
(126/89/18)	sex (M)	−0.15	0.16	−0.98	0.329
	recapture (yes)	0.07	0.14	0.48	0.629
	**sex (M):recapture (yes)**	**0**.**39**	**0**.**19**	**2**.**1**	**0**.**036**
**established**	intercept	1.51	0.18	8.42	<0.001
(63/53/6)	sex (M)	0.24	0.2	1.16	0.247
	recapture (yes)	−0.05	0.26	−0.21	0.833
	sex (M):recapture (yes)	0.21	0.3	0.69	0.494
**activity (binomial)**					
**males**	intercept	0.27	0.4	0.63	0.526
(161/101/21)	colonization zone (edge)	−0.27	0.47	−0.56	0.573
	recapture (yes)	0.46	0.36	1.29	0.197
**females**	intercept	−0.44	0.56	−0.77	0.439
(129/98/23)					
	colonization zone (edge)	1	0.65	1.56	0.118
	recapture (yes)	0.56	0.45	1.23	0.291
**edge**	intercept	0.068	0.35	1.94	0.052
(181/127/120)	**sex (M)**	**−0**.**78**	**0**.**37**	**−2**.**1**	**0**.**031**
	recapture (yes)	0.64	0.38	1.7	0.090
**established**	intercept	−0.5	0.45	−1.13	0.259
(109/75/7)	**sex (M)**	**0**.**91**	**0**.**42**	**2**.**14**	**0**.**032**
	recapture (yes)	0.25	0.44	0.575	0.565

Behaviour of bank vole females did not differ between zones (*z* = 1.0, *p* = 0.320; *post hoc*, table 3).

With repeated testing, male voles at the edge increased their exploration of the exposed area (number of crossings: *z* = 3.5, *p* > 0.001), while males in established populations did not (all *z* < 0.8, *p* > 0.407, interaction of zone and repeated testing, table 2). Exploration also increased with repeated testing at the expansion edge (figure 1*d*, table 2 and *post hoc* tests, table 3, *z* = 2.1, *p* = 0.038), but not in established populations (*z* = 1.3, *p* = 0.185, interaction of factors zone and repeated testing, table 2).

Wood mice behaviour did not differ between the colonization zones of bank voles or the pre-arrival zone (electronic supplementary material, table S4). The test experience (repeated testing) of an individual mice increased its probability to emerge, number of crossings and activity, independent of the colonization zone (electronic supplementary material, table S4).

## 4. Discussion

In our study on non-native bank voles in Ireland we found that male bank voles at the expansion edge exhibited more timid, risk-averse behaviour compared to those in the established populations. Furthermore, males at the expansion edge habituated to repeated testing by becoming bolder, compared to the behaviour of their consexuals at the established ranges, and also compared to females. Finally, wood mice showed no behavioural shifts across colonization zones. Since wood mice and female bank voles did not differ along the expansion gradient, we attribute the observed differences in behavioural responses of male bank voles to the expansion process of the population, and not to bioclimatic gradients or local site differences (table 1 and electronic supplementary material, S1). Surprisingly, although facing similar challenges of unpredictable environments, non-native bank voles in this study had not developed bold phenotypes, as was shown earlier for forest rodents in urban environments (e.g. [3,31]) compared to their rural reference populations, further indicating the different nature of urbanization and range expansion processes.

While male fish and birds appear to be more aggressive at the expansion edge [16,18] the male non-native rodents observed here were more timid and risk-averse at the expansion edge, compared to those in established populations. Rodents are a taxon under high predation pressure, and have evolved to a rather hidden lifestyle, avoiding open spaces and dangerous daytimes, which seem to favour increased cryptic and timid behaviour during a colonization process. Many small rodent populations undergo population fluctuations with dramatic regional population crashes at times (e.g. [49]) and may thus have a pre-adaptation to re-colonization processes. At the expansion edge, male voles were more flexible than at established populations, as indicated by stronger habituation to repeated testing. Rodents are able to adjust to human-made changes in their environment (e.g. urbanization [3,50]) and challenging environments by increasing their behavioural plasticity at both the genotypic and phenotypic level. A previous study of the bank vole colonization of Ireland found significant enrichment for genic SNPs (single nucleotide polymorphisms) to correlate with distance from the site of introduction, including genes ‘that could influence behaviour and a gene involved in sexual differentiation’ [20], which was confirmed at the phenotypic level by the present study. Furthermore, behavioural plasticity is related to risk taking behaviour in bank voles [51] and our results show that both are relevant traits in dispersal processes. The ability of a species to adjust to novel challenges is likely to contribute to its ultimate success in novel environments.

Sex differences operated in the opposite direction in the two zones: at the expansion edge, male bank voles were more risk-averse and less explorative compared to females, while in established populations, males were more risk-prone and more explorative. Behaviour of female bank voles did not differ between zones. In most mammal species, males are the dispersing sex [33,34,52]. Avoiding risks may be a trait that is under strong selection in the expansion process of a population while individuals disperse into areas void of conspecifics and may thus be expressed most strongly in the dispersing sex. With spatial selection for dispersal ability along the edge of an expanding population, populations get spatially sorted by individuals' dispersal abilities over generations. This may cause a runaway evolution of dispersal at the invasion front [53,54]. A strong link of spatial behaviour to behavioural traits [9,25], with shy and thorough explorers using larger areas, may then explain measured differences in behavioural traits of male voles between established and edge populations.

Our findings could have implication on the management of invasive species, among which rodents are a very successful taxon globally. Management approaches including bait uptake and trap entry rely on the propensity of animals to take risks and approach novel objects or novel food, and may thus be less successful in edge populations featuring shy and careful phenotypes, illustrating the need for behaviour-based management [55] of invasive populations at different stages of biological invasions. As rodent behaviour can also determine transmission levels of rodent-borne disease to humans [56] our findings can be expected to have implications on disease transmission. This is of particular concern given the predicted expansion of rodent populations in temperate regions under climate change scenarios [57] in urban areas.

Wood mice behaviour did not differ between the colonization zones of bank voles or the pre-arrival zone. Our results on mice strengthen the results of parallel tests on voles, showing that there are no effects of environmental or temporal variables, which would have affected both species, except geographic location (i.e. colonization zone in voles). Population sizes of wood mice are negatively related to the increasing populations of invading bank voles [38,39]. A lack of behavioural differentiation by mice in response to voles’ presence allows several interpretations. Wood mice, bank voles and other forest rodents are sympatric throughout Europe, while in Ireland, prior to the introduction of the bank vole, wood mice were the only forest rodent and occur at higher density than in continental Europe. Apparently, when mice arrived in Ireland a millennium ago [35], release from heterospecific competitors was compensated by conspecific competition. Since in this study behavioural adaptations of mice to the re-appearance of competitors were not observed, in contrast to other competition systems [36] competitive heterospecific release may not have affected wood mice behaviour. Furthermore, boldness, as measured here, may rather be affected by predation risk than by competition. In addition, crepuscular and nocturnal mice may not reveal behavioural adaptations when studied during bright daylight. Lastly, voles and mice are partially segregated in their daily activity patterns, potentially decreasing strong behavioural interference. Numerical competitive effects of voles on nocturnal mice may mainly function via indirect resource depletion, with voles consuming similar food resources to mice, but are able to deplete resources at both day and night.

To conclude, our study confirmed the importance of a behavioural invasion syndrome at the edge of the vole's expansion range. Non-native bank voles in Ireland were more cautious at the expansion edges of the species distribution and habituated faster in repeated tests compared to voles in established populations. We suggest that the timid dispersal strategy of colonizing rodents at the expansion edge may explain why rodents worldwide are a highly successful, invasive taxon. Results may have implications for the management of invasive species, requiring different measures at expanding populations compared to established ranges.

## Data Availability

The data that support the findings of this study are available on Dryad Digital Repository: https://doi.org/10.5061/dryad.fbg79cp15 [59]. Additional information is provided in electronic supplementary material [60].
